# Extending Integration: Interventions Supporting Communication and Collaboration Between Patients with Neurological Diseases, Their Informal Caregivers and Healthcare Staff – a Scoping Review

**DOI:** 10.5334/ijic.8577

**Published:** 2025-01-28

**Authors:** Eskil Degsell, Lina Al-Adili, Petter Gustavsson, Mats Brommels, Petra Dannapfel

**Affiliations:** 1 Swedish Brain Tumour Association, Sweden; 2 Neuro-Oncology Clinical Research, Innovation, Implementation and Collaboration, Karolinska University Hospital, Stockholm, Sweden; 3 Department of Microbiology, Tumour and Cell Biology, Karolinska Institutet, Stockholm, Sweden; 4 Medical Management Centre, Department of Learning, Informatics, Management and Ethics, Karolinska Institutet, Stockholm, Sweden; 5 Division of Psychology, Department of Clinical Neuroscience, Karolinska Institutet, Stockholm, Sweden; 6 Department of Health, Medicine and Caring Sciences, Linköping University, Linköping, Sweden; 7 Hospital Group West, Western Gothia Healthcare Region, Gothenburg, Sweden

**Keywords:** integrated care, person centred care, neurological diseases, cognitive impairments

## Abstract

**Introduction:**

Addressing challenges due to demographic changes and the quest for improved value in healthcare requires an extended integrated approach to care that fosters collaboration between all stakeholders, especially within collaboration supporting cognitively impaired patients. The aim is to review existing studies on interventions to improve communication and collaboration between such patients, their caregivers and healthcare staff.

**Methods:**

Following PRISMA guidelines, we systematically searched electronic databases Medline (OVID), CINAHL (Ebsco), and Web of Science (Clarivate) for peer-reviewed literature [2010–2020] focusing on intervention studies. Papers were excluded if not assessing the impact of interventions or only presenting a study protocol.

**Results:**

Twelve studies explored diverse approaches to social support, all with the aim of improving communication and collaboration among stakeholders, and identified three intervention types: *supporting empowerment, promoting collaborative disease management, and coping*, and *enhancing communication and relationships*.

**Discussion:**

The interventions employed various approaches and assessed a range of outcomes, demonstrating the benefits of enhancing communication and collaboration among stakeholders. Yet only a few studies included the full triad of partners in care.

**Conclusion:**

There is still much to be done to achieve the extended integration of care services and support that will benefit from patient and caregiver involvement.

## Background

Healthcare systems are challenged by rising healthcare costs due to an ageing population and new treatments [13,14]. Demographic changes will lead to an increase in people with long-term chronic conditions, with an increased burden on healthcare and society at large [15]. At the same time, the introduction of “value-based healthcare” [16] has highlighted the importance of having improved outcomes as part of the overarching management strategy. There is ample evidence that the active participation of patients in designing, implementing and monitoring care will improve patient outcomes, well comparable to the impact of medical interventions [17,18]. One important way of addressing these challenges is better coordinated care which reduces duplication of services provided and cost-driving waits [19,20]. Consequently, integrated care is seen as a key strategy in health systems around the world striving to improve their performance [19]. Valentijn et al highlights the importance of the seamless linking of services and interprofessional partnerships to achieve integration [21]. We focus in the following on *clinical integration* where the patient pathway is coordinated across staff and different organisations [22].

Kee et al state that integrated care requires “effective collaboration between actors with different backgrounds” which depends on the behaviour of these actors. Using insights from organisational psychology they identify behaviours that promote collaboration. Those are that actors speak up about their interests and perspectives, listen to the others and process the information received [23]. Consequently, communication between actors is a central activity required for collaboration. Fox et al studied communication practices in primary care to understand collaborative activities and created a typology of those observed. Three types emerged: communication related to coordinating activities, assisting other’s sense-making and working to understand together [24]. We, therefore, in this study focus on communication and collaboration practices between a triad of actors, patients, next-of-kin (caregivers) and health professionals that can promote integrated care. Although the intention was not to perform a theory-guided study, we seek to deepen our knowledge of those elements of integrated care and find that these concepts from organisational psychology provide useful insights.

We represent a research team with members (clinicians, researchers and caregivers), brought together in an ambition to improve care for brain tumour patients. We soon realised that also other neurological diseases often negatively impact the patient’s perceptions, cognition, mobility, and emotions [25,26,27,28]. Cognitive deficits increase as the disease progresses leading to increased needs of support and care [44,45]. Further, cognitive deficits affect patients’ quality of life and have a profound impact on their relationships with family and friends, resulting in the possible development of a variety of crisis trajectories [44,45]. People with cognitive impairment often experience challenges in their social relationships with family or friends [46,47], affecting quality of life [48,49,50,51,52,53,54]. Being diagnosed with a neurological illness raises many concerns for patients and caregivers related to the progression of the disease, that can lead to poor psychological well-being and reduced health-related quality of life [36,38,39,40].

While individuals with neurological diseases often maintain long-term relationships with healthcare staff and depend on informal caregivers for their care [25,26,27,28], there is to date a limited understanding of how to assess and improve communication and collaboration among this triad of key stakeholders. This is necessary for promoting an extended integrated approach to care that incorporates a person-centred perspective [13,29,30]. This will result in an increasing complexity of care arrangements and involve more stakeholders in care, calling for better coordination and integration of care services [31].

The current status of integrated care in neurology was recently explored by Feng et al [32] in a narrative review of studies in seven neurological conditions. Those disease-specific models varied, often highlighting technological care solutions, patient education and empowerment, the involvement of care coordinators, the design of individualised care plans, vertical integration of clinical services and care pathway planning. When analysing these studies, the authors identified heterogenous definitions of healthcare integration. Whilst some models included support for self-management, many of the definitions emphasised multidisciplinary care but lacked a more comprehensive approach including a focus on active patient participation. Families are mentioned but the concept of the care triad in contrast to the dyad is not introduced. As improvements the authors suggest that integrated care models in neurology should include the alignment of patient and provider expectations. Those expectations, expressing needs and goals, should be actively monitored and assessed by validated outcome measures, including patient-reported measures, quality of life, functional independence, caregiver burden and patient, caregiver and clinician satisfaction [32].

Integrated health arrangements are expected to have a positive effect on the continuity of services, efficiency, quality, and access to care, and benefit especially people with chronic conditions [19,33,34]. However, in practice there is a tendency that focus on integration takes a disease focused perspective rather than a person-centred [13,29,30]. This is related to how “care” is defined. For professionals the clinical perspective on care dominates. For patients and families “care” might be more holistic, incorporating also social dimensions and a more general concept of wellbeing [13,35].

In Europe one of three persons is active as caregiver [36]. A study from Sweden estimated the value of informal care to 3% of the GDP, a substantial addition to formal care consuming a GDP share of 12% [37]. This informal care as provided by family members, partners, children, and friends is growing rapidly [36,38,39,40]. Greater emphasis on outpatient care for patients with cognitive impairments will mean that the responsibility of family members will increase [41]. A study in Canada by Lewanczuk [2021] found that 90% of care provided was self-care or informal care [30]. Providing essential support and assistance is crucial for those who care for these patients [25,26,27,28]. The impact of caregiver involvement goes beyond support. The level of caregiver mastery was associated with improved survival among glioblastoma patients [42]. This shows how important it is that cooperation and communication activities also aim at increasing patient and caregiver competence and confidence [43].

Relationships, communication and collaboration are a foundation for successful and integrated care between all involved – patient, caregiver and health professionals [13,29,30,44,45]. As demonstrated above, there is to date limited knowledge about interventions that address these aspects in the care of patients with neurological diseases and few studies evaluating the impact of those for all involved stakeholders. Hence, this scoping review aims to identify interventions focused on improving communication and collaboration between the triad of patients with neurological diseases, healthcare staff and caregivers as well as the impact of those interventions.

## Methods

As a preliminary exercise, a rapid review of published and grey literature on interventions to improve collaboration and communication between patients, healthcare staff and caregivers was performed to gain a better understanding of relevant concepts and terms and assess how these constructs are currently defined or described. Informed by this review, a panel of key experts, representing nursing, oncology and neurology, patients, caregivers, managers, and researchers agreed on what interventions to focus on in the study. The review showed that the specific topic of improving communication and collaboration among these three stakeholders is under-researched. Only few neurological diagnoses with cognitive impairment were identified among those studies.

In order to explore that topic systematically and comprehensively we decided to conduct a scoping review with the aim to identify specific interventions adopted to improve communication and collaboration between patients, caregivers and health staff and their possible impact. This approach was chosen as a scoping review is especially suitable to explore under-researched areas, identify knowledge gaps, scope a body of literature and to clarify concepts and to guide possible future systematic reviews [49]. This was done using the five-step framework presented by Arksey and O’Malley [50] and the Preferred Reporting Items for Systematic Review and Meta-Analysis (PRISMA-SCR) statement was applied to ensure rigour in reporting the methodology [51].

### Step 1: Identifying the research question

Based on the discussions with the panel members referred to above the following research questions were formulated, applying the PICO framework [52]:

1. What communication and collaborative activities were included in the interventions (I) involving patients, caregivers and health professionals (P)?
2. What was the aim of the interventions?
3. What impact of the interventions have been reported (O)?

When extracting information from the selected articles the study design was registered, including that of a possible comparative approach (C).

### Step 2: Identifying relevant studies

The search strategy was iteratively developed by the research team with the aid of an experienced medical librarian between September and November 2020. Searches was made in November in three electronic databases: Medline (OVID), CINAHL (Ebsco) and Web of Science (Clarivate). The search query was first developed for Medline (Ovid interface). This search strategy was modified as required to be used with CINAHL and Web of Science. Key search terms included Care map, Professional-Patient Relations, Patient-Centred Care, Professional-Family Relations, Shared Decision Making, Cooperative Behaviour, Interaction, Network, Partnership, Relation, Support, Care Plan, Co-care, and Patient-Focus. The disease groups to be included were specified by diagnosis.

### Step 3: Inclusion/exclusion criteria and study selection

Inclusion criteria were:

- Integrated care interventions focusing on the following diagnosis: Amyotrophic Lateral Sclerosis, Dementia, Huntington’s Disease, Brain Neoplasms.
- The interventions shared activities that include a minimum two of the actors of interest (patient, caregiver, healthcare staff)
- The article reported measured outcomes.

Exclusion criteria were:

- The article reported a Study protocol only
- The intervention addressed manifestations of disease but not the relationship between patients, caregivers and providers.

The search included English-language studies published in peer-reviewed journals in the years 2010–2020 and was not limited to country of origin or setting. After the initial searches of the databases were completed, the research team reviewed the first 100 titles from each electronic database to ensure that the most appropriate search terms and strategy had been used. In addition, the panel of key experts in integrated care were consulted about publications they considered relevant to include in the review. The search strategy was modified accordingly, and the term Implementation was added to the final search string, which is exhibited in Supplementary File 1. All study designs were included since scoping reviews do not examine study quality [50].

In a first phase of the selection process titles and abstracts of publications were read independently by two members of the research team and deemed eligible if inclusion criteria were met. In the second phase full texts were read and assessed by all authors. The articles selected were imported to the Rayyan web tool for further analysis [48].

**Figure 1:**
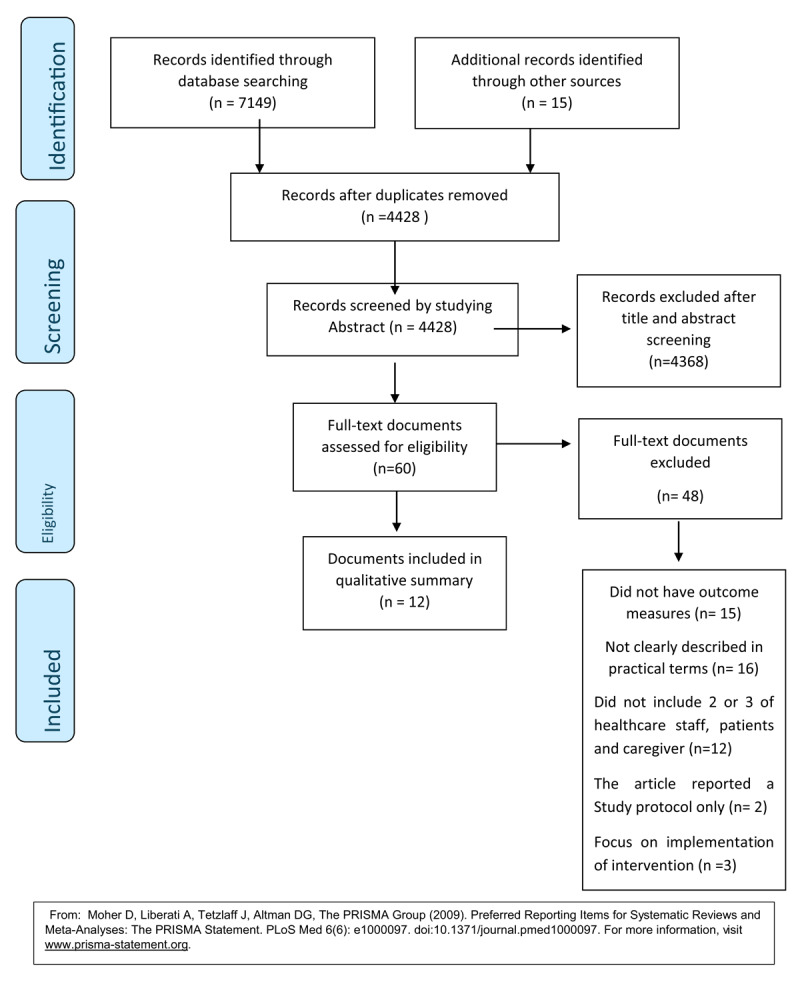
The PRISMA flow diagram illustrating the database searches, abstracts screened, and full texts retrieved in the scoping review.

A total of 4,428 articles, which included the 15 outside the search that were proposed by the experts, underwent title and abstract review which resulted in 4,368 articles being excluded by applying the exclusion criteria. Following the first screening, 60 articles were included for full text review and were read and discussed by the authors applying the criteria strictly. Of those 60 articles 48 articles were found not to meet the inclusion criteria. The remaining 12 were re-read and consensus was reached to include these twelve in the final sample for data extraction and analysis. Figure 1 provides an overview of the screening and abstraction process.

### Step 4: Data extraction and analysis

The twelve articles were read in full by two of the authors and subjected to directed thematic analysis, following the guidelines of Braun and Clarke [53] in order to capture the content and implementation of the interventions as guided by the research questions. A data extraction tool was developed and refined by all authors using the descriptive-analytical method described by Arksey and O’Malley [50]. The chart designed was divided into two sections. First, an overview was provided that summarises basic information of the publication and its content as guided by the research questions. The overview section covers names of the authors, publication date, country where the study took place, main objectives of the study, how the study was conducted and the setting of the study. In the second section content corresponding to the research questions with specifications were summarised and compiled into four columns. The first column lists activities of collaborative care to build and strengthen communication and collaboration between patients, caregivers, and healthcare staff to achieve better health outcomes. The second column names stakeholders involved. In the third column the way in which interventions were delivered are specified. In the fourth column outcomes and lessons learned are reported. The extraction sheet thus includes reported collaborative activities and core components of the interventions, targeted stakeholders, intervention delivery methods, and reported results. These steps are based on several methodological recommendations for scoping reviews [50,55]. The data extraction sheet, the column headings of which guided the content analysis, is presented in Supplementary File 2.

## Results

Of the twelve papers nine reported quantitative studies and three employed qualitative methods [4,8,9]. Most of the studies primarily centred around dementia [1,2,3,4,5,6,8,9,11], while the remaining studies addressed brain tumour and Huntington disease [7,10,12]. Most of the studies focused on patients and caregivers [1,2,3,5,6,7,9,11,12], while three included healthcare staff [4,8,10]. Few interventions were conducted remotely [1,6,12], using web-based interventions or telephone, whereas the rest mainly consisted of physical meetings. The duration of the analysed interventions varied between a couple of weeks to months.

Three categories of interventions were identified: a) *supporting empowerment*; primarily focused on empowering participants to enhance their self-care capabilities and improving caregivers competence b) *promoting collaborative disease management and coping*; aiming at strengthening caregivers in their role and providing support to patients in coping with their disease, and c) *enhancing communication and strengthening relationships*; fostering improved communication among stakeholders, along with promoting collaborative decision-making.

**Figure 2:**
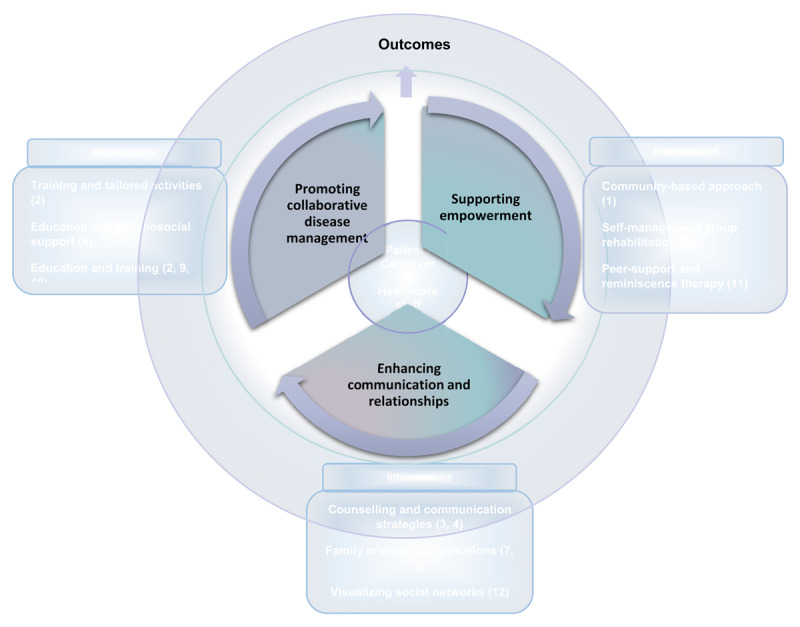
Overview of interventions aimed to support communication and collaboration among patients with neurological diseases and cognitive impairments, caregivers, and healthcare staff.

Acknowledging the interconnectedness between intervention categories, Figure 2 illustrates the findings and the focus of each category with descriptions of the intervention types. Patients, caregivers and healthcare staff are at the centre collaborating to achieve various outcomes.

### 1 Supporting empowerment

Interventions aiming to empower patients and caregivers included volunteering opportunities [1], peer support [11] and self-management skills development [5], all tailored to the individual needs, abilities, and preferences of patients and their caregivers. These interventions mainly assessed outcomes as *quality of life* as well as *cognitive functioning, neuropsychiatric symptoms, emotional impact, care burden, competence, and costs of care*. See Table 1 for detailed information.

**Table 1:** Summary of findings on interventions supporting empowerment and assessed outcomes.


FIRST AUTHOR	INTERVENTION	DESCRIPTION OF INTERVENTION	PARTICIPANTS	EXAMPLES OF ASSESSED OUTCOMES

*Dröes* [1]	DementTalent Dementcoach STAR e-learning	Volunteer work for patients, Telephone coaching and one-line course for caregivers	Patients with Dementia and caregivers	Moderate positive effect on neuropsychiatric symptoms of patients. Caregivers reported less emotional impact and increased happiness compared to those without. No differences in QoL, burden, and competence for patients and caregivers

*Charlesworth* [11]	Carer Supporter Programme [CSP] Remembering Yesterday, Caring Today [RYCT]	Peer-support and reminiscence therapy	Patients with Dementia and caregivers	Improved caregivers’ perceived relationship with patients. No significant improvements in QoL for patients or informal caregivers

*Laakkonen* [5]	Self-management group rehabilitation	Develop self-management skills and self-efficacy.	Patients with Dementia and caregivers [spouses]	Beneficial effects on QoL of spouses and cognitive function of patients. No increase in total costs of care.


#### 1.1 Self-management group rehabilitation

Self-management group rehabilitation is an approach described to enhance patients’ abilities, self-care skills, and capabilities, along with their caregivers [5]. The intervention encouraged participants to share ideas and find solutions to everyday problems. The intervention consisted of four-hour group sessions at a day centre once a week for eight weeks. Good group dynamics and mutual trust were essential in improving these self-management skills. The intervention showed beneficial effects on the quality of life of spouses and cognitive function of patients without additional costs.

#### 1.2 Peer-support and reminiscence therapy [11]

These interventions were evaluated separately and in combination, compared to standard care, for individuals with dementia, conducted over several months, with multiple sessions for both. The trial demonstrated measurable benefits in improving relationships between with patients and caregivers, but no significant effects on quality of life.

#### 1.3 Community-based approach

Dröes et al. conducted three interventions [1]. *DemenTalent* involved patients with dementia volunteering in a community, based on their talents and interests [1]. *Dementelcoach* offered telephone coaching to caregivers, while *STAR e-Learning* provided a course to increase caregivers’ knowledge and skills in person-oriented dementia care. *DemenTalent* showed a moderate positive effect on the neuropsychiatric symptoms of persons with dementia and reduced the emotional stress of caregivers. However, no differences were found in caregiver burden, sense of competence, or overall quality of life in comparison with a regular support programme.

### 2 Promoting collaborative disease management

The interventions in this category focused on enhancing collaborative disease management by providing education and skills training to patients and their caregivers [2,6,9], including healthcare staff in one study [10]. These interventions assessed various outcomes including *behavioural symptoms, caregiving mastery, emotional health strain, dyadic relationship strain, coping, role captivity, anxiety and depression, and care burden*. See Table 2 for detailed information.

**Table 2:** Summary of findings on interventions promoting collaborative disease management and assessed outcomes.


FIRST AUTHOR	INTERVENTION	DESCRIPTION OF INTERVENTION	PARTICIPANTS	EXAMPLES OF ASSESSED OUTCOMES

*A’Campo* [10]	Patient Education Program for Huntington’s disease [PEP-HD]	Education and training-developing coping strategies	Patients with Huntington Disease, caregivers, and healthcare staff	Patients improved in behavioural symptoms, anxiety, coping style, and received more social support. Caregivers experienced reduced psychosocial burden.

*Judge* [9]	Acquiring new skills while enhancing and remaining strengths [ANSWERS]	Educational and cognitive rehabilitation skills training	Caregivers Patients with Dementia	Improved well-being among caregivers. Reduced emotional health strain, relationship strain, and role captivity. Fewer symptoms of anxiety and depression.

*Mavandadi* [6]	Individualized dementia care management [CM] Telehealth Education Program [TEP]	Management education and telephone-based collaborative care program for problem-solving and coping skills training	Patients with Dementia and caregivers	Reductions in caregivers’ negative reactions to behavioural symptoms. Improvements in coping and caregiving mastery.

*Novelli* [2]	Tailored Activity Program—Brazilian version [TAP-BR]	Matching activities to patients’ capabilities and preferences and training for informal caregivers to manage the progression of dementia.	Patients with Dementia and caregivers	Perceived improvement of QoL among caregivers but not patients. Positive impact on caregivers’ distress. No reduction in overall care burden


#### 2.1 Education and training

The education programmes aimed at increasing patients’ and caregivers’ understanding of the disease, its symptoms, and management strategies in order to support informed decision-making and self-management. The *Patient Education Program for Huntington’s disease* involved all three stakeholders and provided training to develop coping strategies [10]. Behavioural symptoms and anxiety were reduced, improving coping, and social support among patients increased. Caregivers experienced reduced psychosocial burden.

Several interventions combined education with skills training [2,9,10], such as the *Acquiring New Skills while Enhancing and Remaining Strengths* intervention for dementia caregivers [9]. Benefits reported were reduced caregiver emotional health strain, relationship strain, role captivity, and improved caregiving mastery.

#### 2.2 Education and psychosocial support

Education combined with psychosocial support was provided through a telephone-based collaborative care management programme for dementia patients [6]. Individualised dementia care offered regular contacts between the caregiver and primary care providers. Caregivers could also to select from a menu of on-line modules covering various topics. Effects were reduced negative reactions among caregivers to dementia-related behaviours and improved coping and caregiving mastery over time.

#### 2.3 Training and tailoring activities

Caregivers were trained to tailor activities to the capabilities of patients and offered support to their caregiving [2]. Although the intervention reduced caregiver distress it did not decrease the overall care burden.

### 3 Enhancing communication and relationships

Training how to communicate and relate to the new situation and changed relationship due to the patient’s illness aimed at fostering understanding among stakeholders [3,4,7,8,12]. Various outcomes were reported such as *rapport and trust building, future care planning, communication skills, effective decision-making, building understanding of patients’ biographies, QoL, connecting with patients, and family functioning*. See Table 3 for detailed information.

**Table 3:** Summary of findings on interventions enhancing communication and relationships and assessed outcomes.


FIRST AUTHOUR	INTERVENTION	DESCRIPTION OF INTERVENTION	PARTICIPANTS	EXAMPLES OF ASSESSED OUTCOMES

*Reblin* [12]	Electronic Support Network Assessment Program [eSNAP]	Web-based application helping caregivers visualize their existing social network resources	Informal caregivers caring for patients with brain tumours	No significant effect on perceived helpfulness of social support or differences in care burden. Reduced depression but anxiety levels remained stable.

*Orosulic-Jeras* [3]	Support, Health, Activities, Resources, and Education [SHARE]	Counselling-based care-planning sessions- assessing patients care values and preferences for future care.	Patients with dementia and informal caregivers	Improved decision-making and future care plans through collaborative care planning.

*Faarup* [7]	Family health conversations [FamHC]	Family health conversations promoting partnership and focusing on the family’s unique perspective.	Patients with brain tumours, informal caregivers and healthcare staff.	No significant effect on QoL, family functioning, and family hardiness in patients and their family members, but suggested potential benefits in later stages of the illness trajectory.

*Kellet* [8]	Family Biography Workshops [FBW]	Workshops to support family and staff to co-construct the history of patients in residential care	Caregivers [dementia] and healthcare staff	Enhanced satisfaction and empowerment for informal caregivers. Improved staff’s ability to connect with patients and manage difficult situations.

*Aasmul* [4]	Advanced Care Planning [ACP]	Repeated communication and decision-making- assesses individual preferences and values, and goals, and potential concerns about care	Patients with Dementia, caregivers, and healthcare staff	Improved communication initially, but challenges in sustaining the gains.


#### 3.1 Counselling and communication strategies

Patients and caregivers were involved in designing and adjusting care plans by staff inviting to continuous, long-term contacts for addressing patient needs and build trust [3,4]. The *Advanced Care Planning* intervention [4] involved early meetings with staff directly after admission and monthly telephone contacts between nursing home staff, patient, and caregivers. The *Support, Health, Activities, Resources, and Education* intervention [3] entailed collaboration with a counsellor to identify patients’ needs and collaboratively create a realistic care plan. Simultaneously, caregivers were helped to understand their loved one’s preferences as disease progresses.

#### 3.2 Family oriented conversations

Family oriented conversations intend to increase understanding between stakeholders and promote social support [7,8]. The *Family Biography Workshops* are designed to help the involved to build a better understanding of the patient’s personal history [8]. Next-of-kin are helped to distance themselves from the caregiver role and staff is able to connect with patients with a new understanding. Family caregivers felt empowered by sharing knowledge that was recognised and valued by participating staff.

*Family health conversations* for patients with glioblastoma multiforme and their family members were evaluated in a controlled study [7]. No significant effects on outcomes were demonstrated but results indicated potential benefits in later stages of the illness.

#### 3.3 Identifying social support

The *Electronic Support Network Assessment Program* aimed to capture and visualise social networks in order to support caregivers [12]. Findings showed that while caregiver burden and distress remained high throughout the study the programme participants experienced significantly less depression than a control group.

## Discussion

This scoping review identified three intervention categories that aim to build and strengthen communication and collaboration between patients with neurological diseases, caregivers, and healthcare staff. *Interventions supporting empowerment* involved self-management support, peer-support, reminiscence therapy and community-based approaches [1,5,11]. *Interventions promoting collaborative disease management and coping* involved education combined with training, psychosocial support, and tailored activities [2,6,9,10]. *Interventions enhancing communication and strengthening relationships* involved counselling and communication strategies, family-oriented conversations, and social support [3,4,7,8,12].

Despite variations in evaluation methods and the different focus of the reviewed interventions, the main findings highlight positive effects of interventions supporting collaborative care on patient outcomes and the well-being of their caregivers, ranging from better symptom management to reduced need for services and, thus, costs of care. Indications of improved quality of life of patients and reduced burden on caregivers and levels of depression were also found. Findings are consistent with previous research showing that social relations and support are important and valuable in conditions entailing cognitive declines [44,45]. Engaging and empowering individuals and their families are critical to person-centred integrated care [13]. Hence, people with neurological diseases require holistic care that extends beyond the healthcare system to encompass the support of their community and family [21,44,45]. Despite research illustrating the great impact of cognitive impairments on a patient’s relationships with their caregivers, and consequently, their overall well-being [44,45], it is evident that this dimension has been in many instances overlooked within the specific diagnostic context we were examining. This underscores the need for more research in this area.

Many articles were excluded from this review since they did not include an evaluation of the intervention nor descriptions of outcomes [n = 124, 30%]. In order to implement and facilitate collaborative healthcare it is crucial to identify and assess the impact of these interventions on all parties involved. Findings highlight the need for more studies that systematically evaluate the effects of these interventions on diverse outcomes, spanning from a micro-level, affecting stakeholders’ well-being to the macro-level, with implications for healthcare organisations and the quality and efficacy of the care given. The few studies that fulfilled the inclusion criteria reflect that the impact of extended integrated care interventions is an underexplored area, particularly in conditions where health deterioration occurs rapidly, such as during cancer treatment [56]. In these cases, effective collaboration is necessary to support patients in adapting to their changing life-situation. However, most of the studies focused primarily on patients with dementia. This might reflect that collaborative care has gained more attention within dementia care, potentially due to the growing elderly population and its associated challenges, requiring innovative solutions [14].

Despite to the ambition of this study to focus on the triad of patients, caregivers and health professionals, only in a few studies the interventions described involved all three parties. This might reflect an existing perception within the healthcare system that the roles of patients and staff should be kept apart. When all three stakeholders were involved collaborative practices like shared decision-making and care planning were part of the programmes [10,3]. More often the focus in collaborative approaches centred on communication and relationships between patients and their caregivers. This shortcoming calls for action as some studies emphasise the value of collaboration among all stakeholders, yielding positive outcomes for all parties involved [57,58]. This collaboration not only benefits patients by improving their outcomes and potentially reducing care costs but also motivates staff in their work and enhances their job satisfaction [57].

The mechanisms behind the positive impact of social networks might be explored through the lens of “collective efficacy”. It applies the concept of self-efficacy – in itself an expression of individual empowerment – to a group. As a group realises what it can achieve through collaborating, this insight will influence the way in which group members act, how much effort they make and their belief in reaching an intended effect [59]. An example of how such collective efficacy can be reached is to be found in end-of-life care. Palliative specialist units have concluded that their care network is incomplete unless it covers care provided by next-of-kin, social care, and, in addition, gains benefits from supportive civic policies and resourcing provided by the community. Those networks are referred to as “compassionate communities”, a term highlighting their ethos [60].

Most of the reviewed studies are quantitative, using standardised scales to evaluate the impact of the interventions. The outcomes evaluated are mainly psychosocial outcomes and fewer included “harder” endpoints, such as mortality, hospitalisation, or costs of care. Deterioration in psychosocial functioning among patients with cognitive impairment has been shown to be a challenge [61,62], which affects patients overall well-being [46] and might explain this choice of outcome measure. Many of these patients experience difficulties connected to their social environment, such as their relationships [44,45]. Some studies suggest an association between psychosocial factors and mortality, claiming that social participation and well-being promotes longevity and health [63]. However, to be able to prove such an association, longer follow-up is needed. None of the reviewed studies conducted extensive long-term exploration into how improvement in psychosocial outcomes affects the efficacy and quality of care in the long-term. Furthermore, the duration of the interventions varied, and some authors proposed that the effect might be more visible in the long-term and not detectable in the short-term [4]. Hence, future research should look beyond short-term results to understand how these interventions can lead to significant changes and improvements in healthcare. Longitudinal studies are also needed to demonstrate long-term effects of these interventions.

While healthcare is advancing in terms of digitalisation and technology use, still only a few studies applied digital or remote approaches. This might be due to the high proportion of dementia studies. The elderly population tends to be less receptive to digital solutions also. In the context of cognitive impairments, maintaining regular contact between patients and healthcare staff is particularly important [64]. However, lack of resources and the low accessibility of care remains a challenge in health care [65]. Remote interventions have been described as one strategy of increasing accessibility of care [66]. The implementation of remote interventions, combined with physical meetings between patients and staff, has been proposed as optimal in improving outcomes. This approach has been shown in a meta-analysis on digital health interventions for people living with dementia and cognitive impairment to promote patient participation in their care [64]. According to this study, these interventions might reduce the risk of injury and hospitalisation and benefit patients who find it difficult to participate in community programmes because of mobility problems, social anxiety or limited physical functioning [64]. Considering these advantages, remote interventions tailored to different patient groups that include collaborative approaches might be one way of improving care and promoting effective communication between stakeholders. Involving caregivers in these collaborative approaches is key, yet the number of digital interventions targeting caregivers is limited to date [66]. Caregivers usually spend more time with patients and have a deeper understanding of the patient’s specific situation. Hence, their participation is necessary to the success of these interventions. To be able to provide continuity, digital interventions focusing on collaboration and communication using resource efficient, evidence-based methods, and using validated standardised methods for evaluating their outcomes are needed.

This study analysed interventions on collaboration and communication engaging the triad of patients, caregivers and health professionals in neurological care. Sullivan defines collaborative practices as those where stakeholders are committed to contribute, their actions are coordinated, shared decision-making takes place and a respectful relationship between all evolves [67]. Focusing on the studies involving the full triad, shared decision-making and coordination in the form of care planning took place [3,4,10]. Those activities were facilitated by training and support by health professionals. Turning to communication, a group rehabilitation programme in dementia [5] encouraged participants to share ideas and find solutions, that is, processes of “speak-up” and “sense-making” could be identified [23,24]. The importance of mutual trust was emphasised [66]. Interventions supporting care planning [3,4] typically focused on clarifying patient needs in order to design realistic plans and building trust, demonstrating “sense-making” and “helping to understand” [23,24]. Family Biography Workshops [8] and Family Health Conversations [7] involved patients, their caregivers and clinicians in series of discussions where patients “spoke up” and told about their lives, enhancing “listening”, helping participants to “process information” and “work to understand together”, thus promoting “sense-making” [23,24]. Although those studies represent a minority of the sample, they provided information that helps us to better understand what behaviours enhance collaboration and communication in the care triad, insights that hopefully will contribute to improving an extended integration of the care for patients with neurological diseases.

The interventions included in this study are heterogeneous, demonstrating a variety of approaches to enhance collaboration and communication between stakeholders. Diverse measures were used to assess the effectiveness of these interventions. This diversity in both intervention types and evaluation methods as well as the few reviewed studies contributes to the challenge of making comparisons and generalising the impact of these interventions. Findings give a limited picture of the interventions in this area although indicate beneficial results. Involving additional diagnoses in the review might have offered a broader perspective. Given this heterogeneity, a systematic review, which would be a recommended next step, would not offer an opportunity to perform a meta-analysis required to provide more generalisable assessments of the outcomes reported in the studies of this scoping review. However, the strength of this study lies in its thorough review process and the rigorous methodology employed, which enhances the overall quality of the research [46,47,49]. The involvement of expert panels in the selection of the studies further increases credibility. The application of thematic analysis facilitated a systematic categorisation of interventions [49,61], with consensus reached within the research group after thorough discussions.

## Conclusions

Findings highlight the positive impact of the reviewed interventions aimed at improving the communication and collaboration between patients with impaired cognition, their caregivers and healthcare staff, ranging from better symptom management to reduced need for services and, thus, costs of care. Indications of improved quality of life of patients and reduced burden on caregivers and levels of depression were also found. Yet only a few studies included the full triad of partners in care. Hence, there is still much to be done in order to reach the extended integration of care services and support that will benefit from patient and caregiver involvement.

## Additional Files

The additional files for this article can be found as follows:

10.5334/ijic.8577.s1 Supplementary File 1. Search string.

10.5334/ijic.8577.s2 Supplementary File 2. Data extraction sheet.
