# Supplementary material for: Extending Integration: Interventions Supporting Communication and Collaboration Between Patients with Neurological Diseases, Their Informal Caregivers and Healthcare Staff – a Scoping Review
Source: Int J Integr Care. 2025 Jan 28;25(1):5. doi: 10.5334/ijic.8577 (PMC11784509; doi:10.5334/ijic.8577)
Supplement: Supplementary File 1. — Search string. [file ijic-25-1-8577-s1.pdf]

Appendix 1

Search string and keywords (inclusion and exclusion criteria)

Databases: **Ovid MEDLINE(R) and Epub Ahead of Print, In-Process & Other Non-Indexed Citations and Daily** 2010 to September 14, 2020
Search Strategy:

#	Searches	
1	exp Professional-Patient relations/	
2	Professional-Family Relations/	
3	Patient-Centered Care/	
4	exp Social Support/	
5	Decision Making, Shared/	
6	Cooperative Behavior/	
7	((adult child* or caregiver* or famil* or kinship* or next in kin or partner* or patient* or relative* or spouse*) adj3 (caregiver* or nurse* or physician* or personnel or professional* or provider* or staff) adj7 (co-product* or collaborat* or compliant* or cooperat* or decision making or interact* or network* or partnership* or relation* or support)).ti,ab,kf.	
8	((adult child* or caregiver* or famil* or kinship* or next in kin or partner* or patient* or relative* or spouse*) adj3 (caregiver* or nurse* or physician* or personnel or professional* or provider* or staff) adj7 (care map* or care plan* or co-care or patient-center* or person-center* or patient-focus*)).ti,ab,kf.	
9	(participatory health or compasionate communit* or peer-to-peer).ti,ab,kf.	
10	or/1-9	
11	Amyotrophic Lateral Sclerosis/	
12	Dementia/	
13	Huntington Disease/	
14	Brain Neoplasms/	
15	"Head and Neck Neoplasms"/	
16	(amyotrophic lateral sclerosis or ALS or charcot disease or dementia or huntington).ti,ab,kf.	
17	((brain or head or intracranial or neck) adj3 (cancer* or neoplasm* or tumor* or tumour*)).ti,ab,kf.	
18	or/11-17	
19	Program Evaluation/	
20	Patient Outcome Assessment/	
21	(implement* or evaluat* or efficien* or effectiv* or assess* or outcome* or intervention* or efficac*).ti,ab,kf.	
22	or/19-21	
23	10 and 18 and 22	

24	limit 23 to (english language and yr="2010 -Current")	
25	limit 24 to (clinical conference or congress or consensus development conference or consensus development conference, nih or editorial or guideline or letter)	
26	24 not 25	
27	("31237659" or "27125270" or "32449496" or "31085663" or "27640872" or "29171960" or "32089513" or "32081097" or "31764053" or "32050779" or "21249954" or "31175194" or "29968510" or "31002025" or "31072861" or "31651321" or "31264707" or "31585057" or "30422754" or "29546325" or "31256612" or "32072866" or "31771566" or "27753612" or "31141133" or "32318261" or "31342861" or "30295037" or "29244125" or "31466807").ui.	
28	26 and 27	
